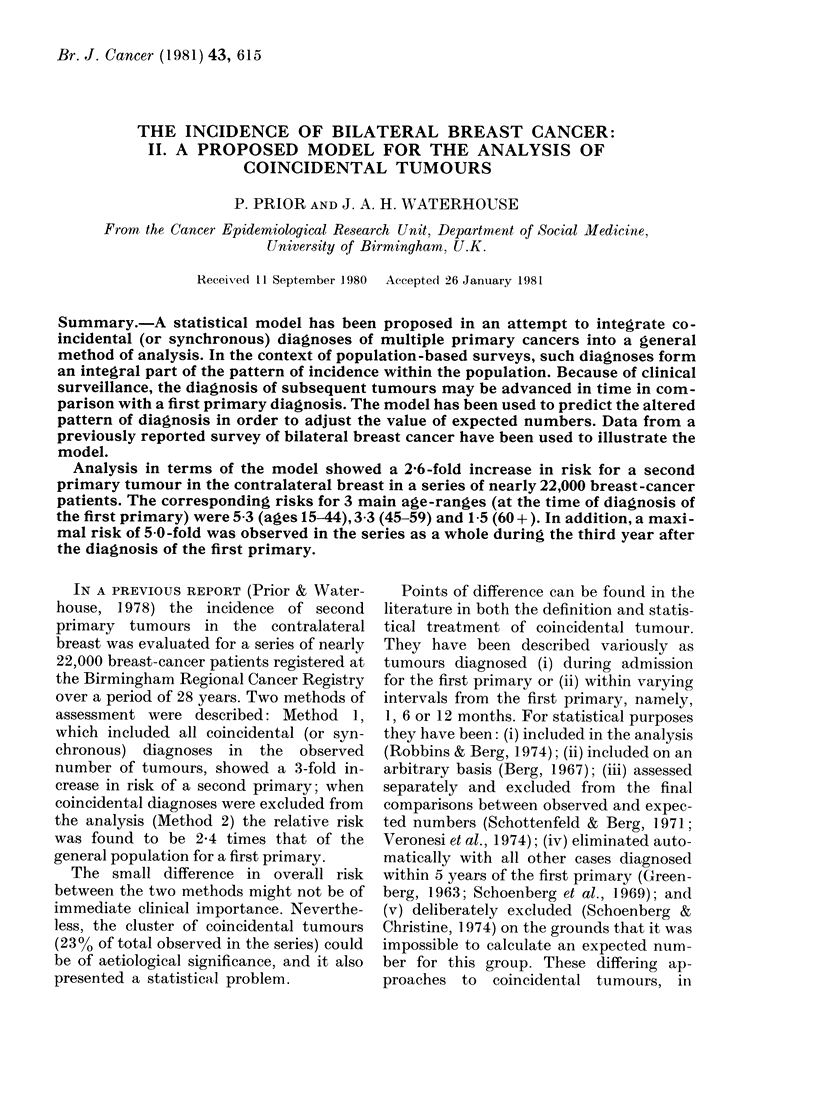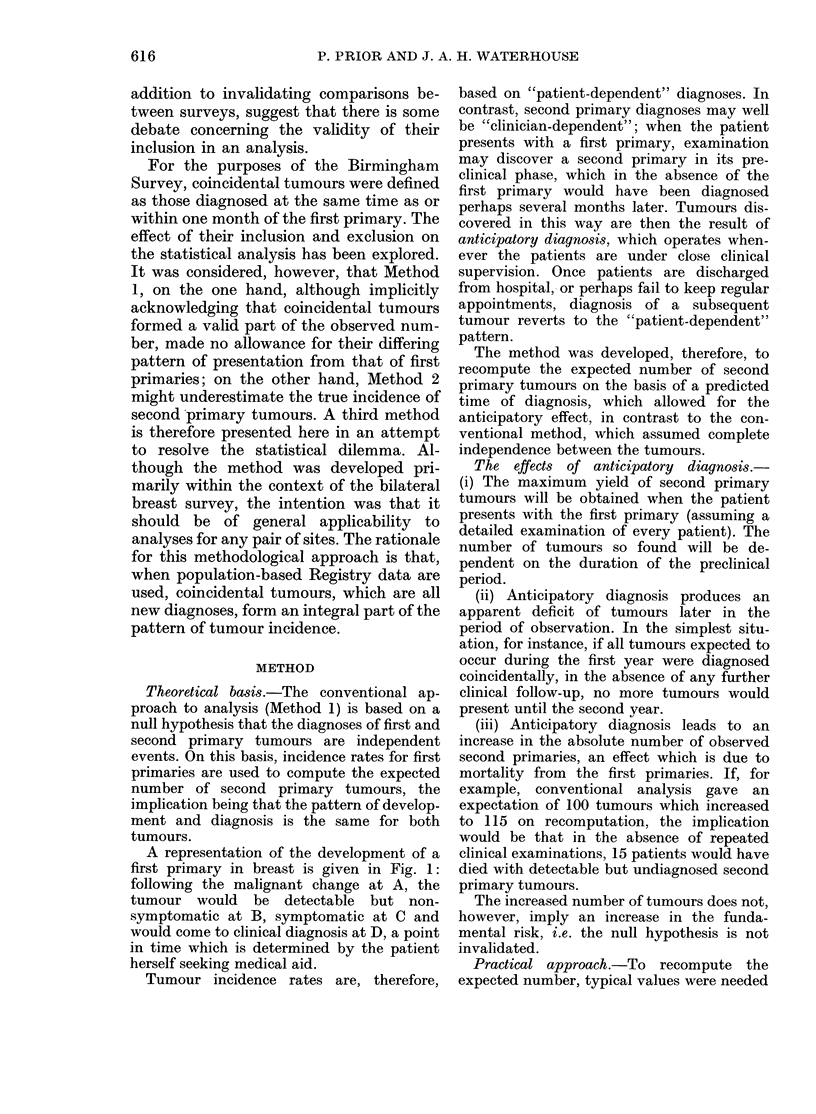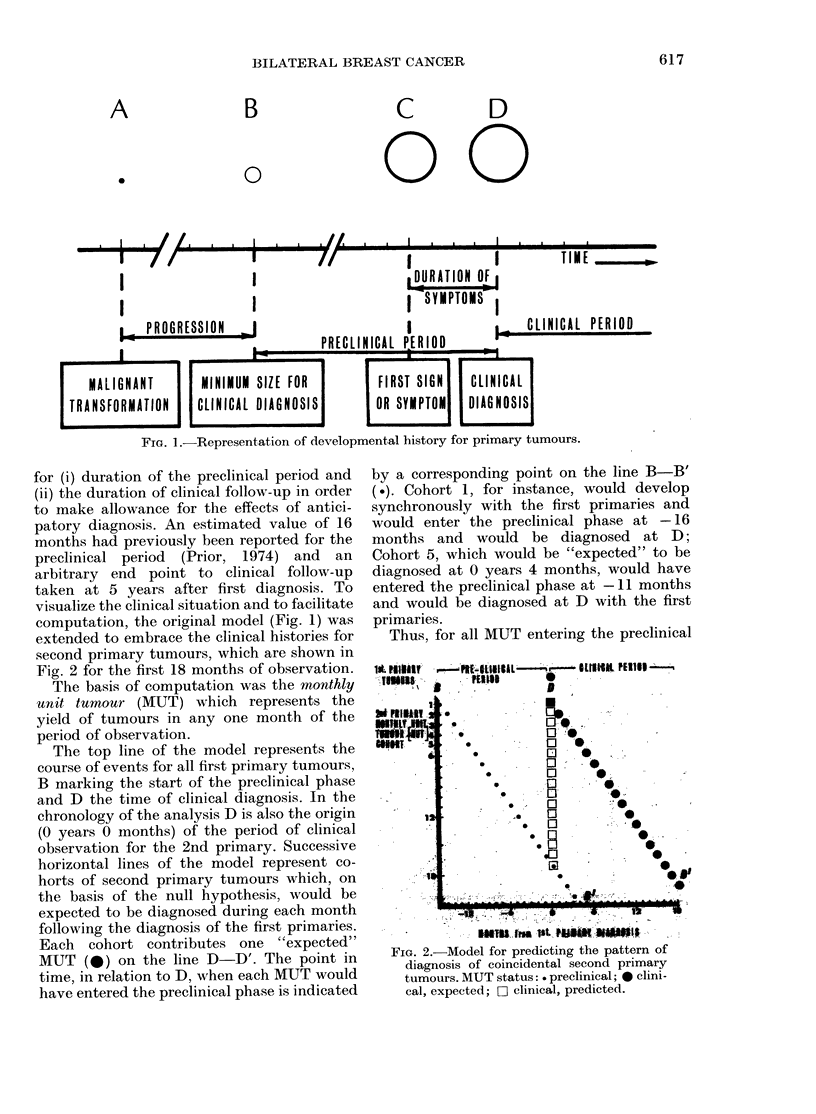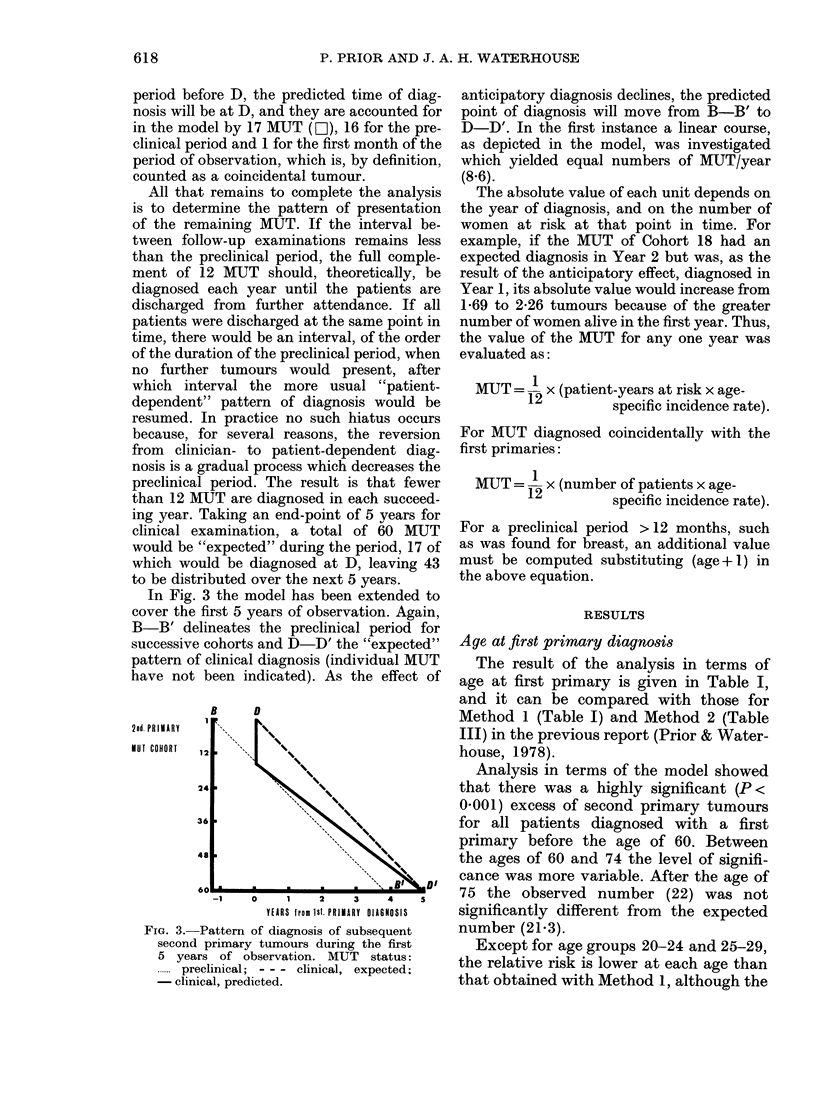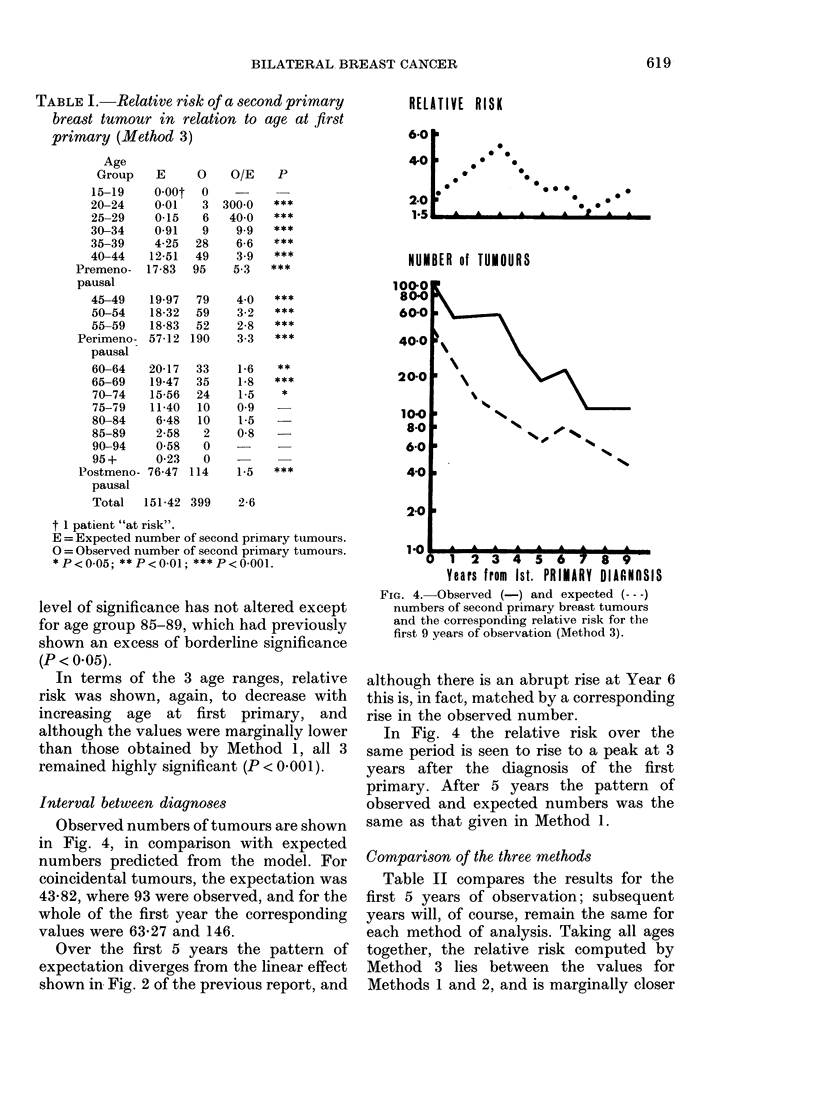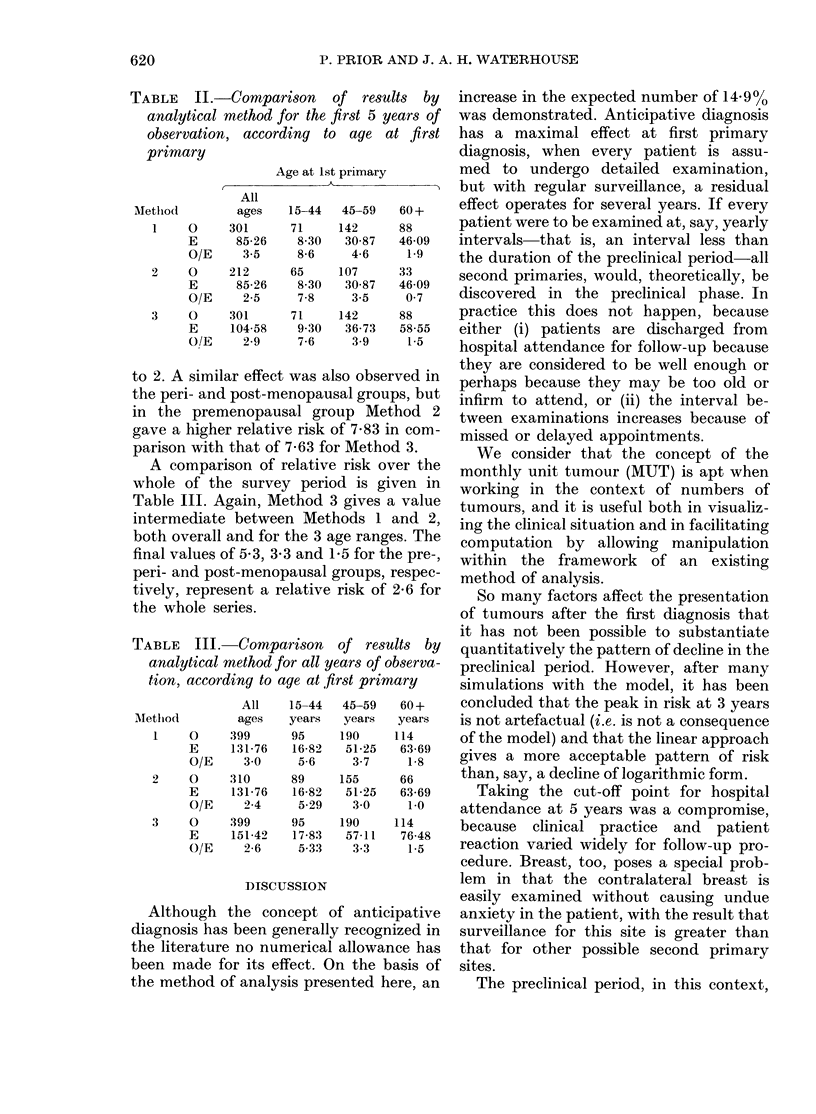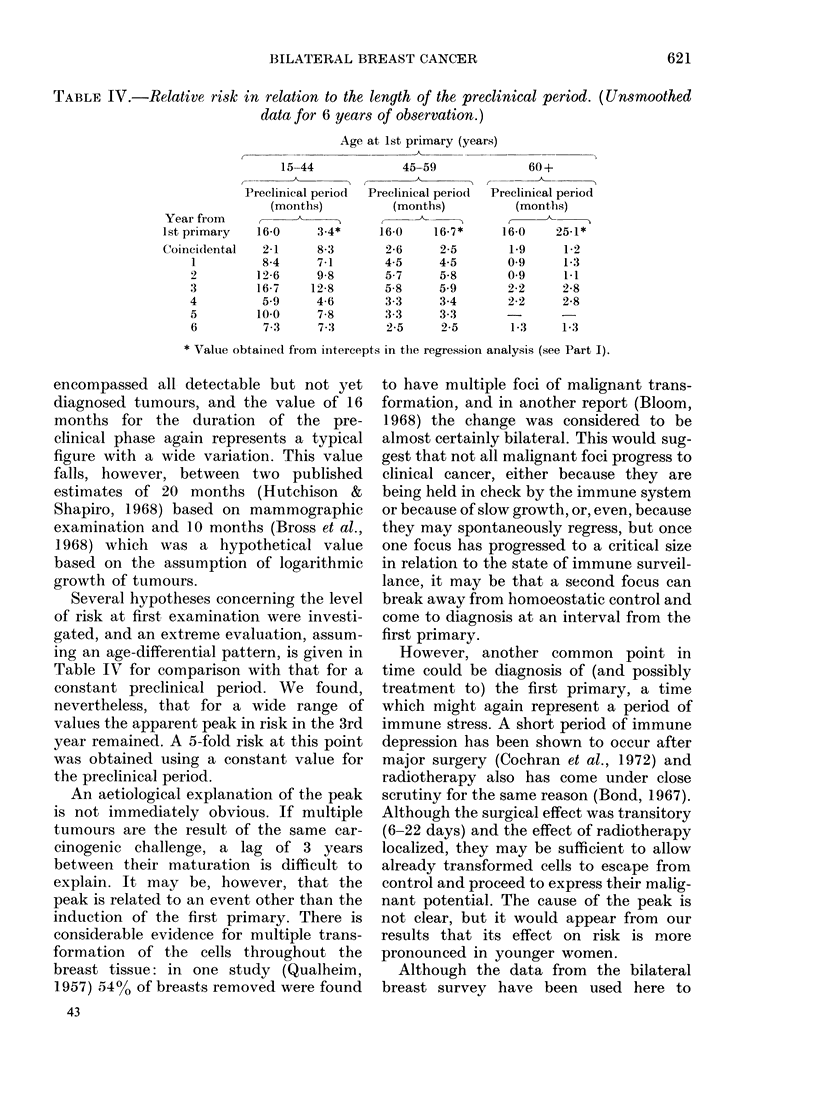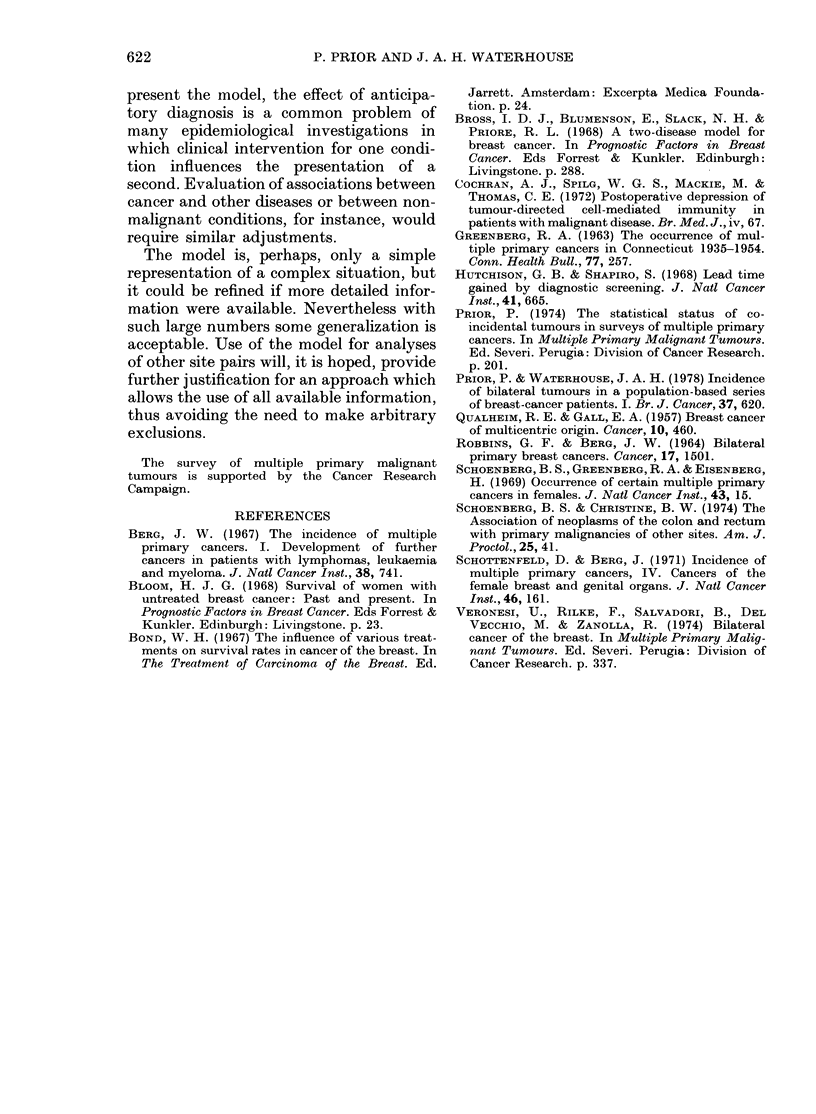# The incidence of bilateral breast cancer: II. A proposed model for the analysis of coincidental tumours.

**DOI:** 10.1038/bjc.1981.91

**Published:** 1981-05

**Authors:** P. Prior, J. A. Waterhouse

## Abstract

A statistical model has been proposed in an attempt to integrate coincidental (or synchronous) diagnoses of multiple primary cancers into a general method of analysis. In the context of population-based surveys, such diagnoses form an integral part of the pattern of incidence within the population. Because of clinical surveillance, the diagnosis of subsequent tumours may be advanced in time in comparison with a first primary diagnosis. The model has been used to predict the altered pattern of diagnosis in order to adjust the value of expected numbers. Data from a previously reported survey of bilateral breast cancer have been used to illustrate the model. Analysis in terms of the model showed a 2.6-fold increase in risk for a second primary tumour in the contralateral breast in a series of nearly 22,000 breast-cancer patients. The corresponding risks for 3 main age-ranges (at the time of diagnosis of the first primary) were 5.3 (age 15-44), 3.3 (45-49) and 1.5 (60+). In addition, a maximal risk of 5.0-fold was observed in the series as a whole during the third year after the diagnosis of the first primary.


*Br. J. Cancer* (1981) **43**, 615

### THE INCIDENCE OF BILATERAL BREAST CANCER: II. A PROPOSED MODEL FOR THE ANALYSIS OF COINCIDENTAL TUMOURS

P. PRIOR AND J. A. H. WATERHOUSE

*From the Cancer Epidemiological Research Unit, Department of Social Medicine, University of Birmingham, U.K.*

Received 11 September 1980 Accepted 26 January 1981

**Summary.—A statistical model has been proposed in an attempt to integrate coincidental (or synchronous) diagnoses of multiple primary cancers into a general method of analysis. In the context of population-based surveys, such diagnoses form an integral part of the pattern of incidence within the population. Because of clinical surveillance, the diagnosis of subsequent tumours may be advanced in time in comparison with a first primary diagnosis. The model has been used to predict the altered pattern of diagnosis in order to adjust the value of expected numbers. Data from a previously reported survey of bilateral breast cancer have been used to illustrate the model.**

**Analysis in terms of the model showed a 2·6-fold increase in risk for a second primary tumour in the contralateral breast in a series of nearly 22,000 breast-cancer patients. The corresponding risks for 3 main age-ranges (at the time of diagnosis of the first primary) were 5·3 (ages 15–44), 3·3 (45–59) and 1·5 (60+). In addition, a maximal risk of 5·0-fold was observed in the series as a whole during the third year after the diagnosis of the first primary.**

IN A PREVIOUS REPORT (Prior & Waterhouse, 1978) the incidence of second primary tumours in the contralateral breast was evaluated for a series of nearly 22,000 breast-cancer patients registered at the Birmingham Regional Cancer Registry over a period of 28 years. Two methods of assessment were described: Method 1, which included all coincidental (or synchronous) diagnoses in the observed number of tumours, showed a 3-fold increase in risk of a second primary; when coincidental diagnoses were excluded from the analysis (Method 2) the relative risk was found to be 2·4 times that of the general population for a first primary.

The small difference in overall risk between the two methods might not be of immediate clinical importance. Nevertheless, the cluster of coincidental tumours (23% of total observed in the series) could be of aetiological significance, and it also presented a statistical problem.

Points of difference can be found in the literature in both the definition and statistical treatment of coincidental tumour. They have been described variously as tumours diagnosed (i) during admission for the first primary or (ii) within varying intervals from the first primary, namely, 1, 6 or 12 months. For statistical purposes they have been: (i) included in the analysis (Robbins & Berg, 1974); (ii) included on an arbitrary basis (Berg, 1967); (iii) assessed separately and excluded from the final comparisons between observed and expected numbers (Schottenfeld & Berg, 1971; Veronesi *et al.*, 1974); (iv) eliminated automatically with all other cases diagnosed within 5 years of the first primary (Greenberg, 1963; Schoenberg *et al.*, 1969); and (v) deliberately excluded (Schoenberg & Christine, 1974) on the grounds that it was impossible to calculate an expected number for this group. These differing approaches to coincidental tumours, in addition to invalidating comparisons between surveys, suggest that there is some debate concerning the validity of their inclusion in an analysis.

For the purposes of the Birmingham Survey, coincidental tumours were defined as those diagnosed at the same time as or within one month of the first primary. The effect of their inclusion and exclusion on the statistical analysis has been explored. It was considered, however, that Method 1, on the one hand, although implicitly acknowledging that coincidental tumours formed a valid part of the observed number, made no allowance for their differing pattern of presentation from that of first primaries; on the other hand, Method 2 might underestimate the true incidence of second primary tumours. A third method is therefore presented here in an attempt to resolve the statistical dilemma. Although the method was developed primarily within the context of the bilateral breast survey, the intention was that it should be of general applicability to analyses for any pair of sites. The rationale for this methodological approach is that, when population-based Registry data are used, coincidental tumours, which are all new diagnoses, form an integral part of the pattern of tumour incidence.

### METHOD

*Theoretical basis.*—The conventional approach to analysis (Method 1) is based on a null hypothesis that the diagnoses of first and second primary tumours are independent events. On this basis, incidence rates for first primaries are used to compute the expected number of second primary tumours, the implication being that the pattern of development and diagnosis is the same for both tumours.

A representation of the development of a first primary in breast is given in Fig. 1: following the malignant change at A, the tumour would be detectable but non-symptomatic at B, symptomatic at C and would come to clinical diagnosis at D, a point in time which is determined by the patient herself seeking medical aid.

Tumour incidence rates are, therefore, based on "patient-dependent" diagnoses. In contrast, second primary diagnoses may well be "clinician-dependent"; when the patient presents with a first primary, examination may discover a second primary in its preclinical phase, which in the absence of the first primary would have been diagnosed perhaps several months later. Tumours discovered in this way are then the result of *anticipatory diagnosis*, which operates whenever the patients are under close clinical supervision. Once patients are discharged from hospital, or perhaps fail to keep regular appointments, diagnosis of a subsequent tumour reverts to the "patient-dependent" pattern.

The method was developed, therefore, to recompute the expected number of second primary tumours on the basis of a predicted time of diagnosis, which allowed for the anticipatory effect, in contrast to the conventional method, which assumed complete independence between the tumours.

*The effects of anticipatory diagnosis.*—(i) The maximum yield of second primary tumours will be obtained when the patient presents with the first primary (assuming a detailed examination of every patient). The number of tumours so found will be dependent on the duration of the preclinical period.

(ii) Anticipatory diagnosis produces an apparent deficit of tumours later in the period of observation. In the simplest situation, for instance, if all tumours expected to occur during the first year were diagnosed coincidentally, in the absence of any further clinical follow-up, no more tumours would present until the second year.

(iii) Anticipatory diagnosis leads to an increase in the absolute number of observed second primaries, an effect which is due to mortality from the first primaries. If, for example, conventional analysis gave an expectation of 100 tumours which increased to 115 on recomputation, the implication would be that in the absence of repeated clinical examinations, 15 patients would have died with detectable but undiagnosed second primary tumours.

The increased number of tumours does not, however, imply an increase in the fundamental risk, *i.e.* the null hypothesis is not invalidated.

*Practical approach.*—To recompute the expected number, typical values were needed

FIG. 1.—Representation of developmental history for primary tumours.

for (i) duration of the preclinical period and (ii) the duration of clinical follow-up in order to make allowance for the effects of anticipatory diagnosis. An estimated value of 16 months had previously been reported for the preclinical period (Prior, 1974) and an arbitrary end point to clinical follow-up taken at 5 years after first diagnosis. To visualize the clinical situation and to facilitate computation, the original model (Fig. 1) was extended to embrace the clinical histories for second primary tumours, which are shown in Fig. 2 for the first 18 months of observation.

The basis of computation was the *monthly unit tumour* (MUT) which represents the yield of tumours in any one month of the period of observation.

The top line of the model represents the course of events for all first primary tumours, B marking the start of the preclinical phase and D the time of clinical diagnosis. In the chronology of the analysis D is also the origin (0 years 0 months) of the period of clinical observation for the 2nd primary. Successive horizontal lines of the model represent cohorts of second primary tumours which, on the basis of the null hypothesis, would be expected to be diagnosed during each month following the diagnosis of the first primaries. Each cohort contributes one "expected" MUT (●) on the line D—D′. The point in time, in relation to D, when each MUT would have entered the preclinical phase is indicated by a corresponding point on the line B—B′ (•). Cohort 1, for instance, would develop synchronously with the first primaries and would enter the preclinical phase at −16 months and would be diagnosed at D; Cohort 5, which would be "expected" to be diagnosed at 0 years 4 months, would have entered the preclinical phase at −11 months and would be diagnosed at D with the first primaries.

Thus, for all MUT entering the preclinical

FIG. 2.—Model for predicting the pattern of diagnosis of coincidental second primary tumours. MUT status: • preclinical; ● clinical, expected; □ clinical, predicted.

period before D, the predicted time of diagnosis will be at D, and they are accounted for in the model by 17 MUT (□), 16 for the preclinical period and 1 for the first month of the period of observation, which is, by definition, counted as a coincidental tumour.

All that remains to complete the analysis is to determine the pattern of presentation of the remaining MUT. If the interval between follow-up examinations remains less than the preclinical period, the full complement of 12 MUT should, theoretically, be diagnosed each year until the patients are discharged from further attendance. If all patients were discharged at the same point in time, there would be an interval, of the order of the duration of the preclinical period, when no further tumours would present, after which interval the more usual "patient-dependent" pattern of diagnosis would be resumed. In practice no such hiatus occurs because, for several reasons, the reversion from clinician- to patient-dependent diagnosis is a gradual process which decreases the preclinical period. The result is that fewer than 12 MUT are diagnosed in each succeeding year. Taking an end-point of 5 years for clinical examination, a total of 60 MUT would be "expected" during the period, 17 of which would be diagnosed at D, leaving 43 to be distributed over the next 5 years.

In Fig. 3 the model has been extended to cover the first 5 years of observation. Again, B—B′ delineates the preclinical period for successive cohorts and D—D′ the "expected" pattern of clinical diagnosis (individual MUT have not been indicated). As the effect of

FIG. 3.—Pattern of diagnosis of subsequent second primary tumours during the first 5 years of observation. MUT status: ...... preclinical; - - - clinical, expected; — clinical, predicted.

anticipatory diagnosis declines, the predicted point of diagnosis will move from B—B′ to D—D′. In the first instance a linear course, as depicted in the model, was investigated which yielded equal numbers of MUT/year (8·6).

The absolute value of each unit depends on the year of diagnosis, and on the number of women at risk at that point in time. For example, if the MUT of Cohort 18 had an expected diagnosis in Year 2 but was, as the result of the anticipatory effect, diagnosed in Year 1, its absolute value would increase from 1·69 to 2·26 tumours because of the greater number of women alive in the first year. Thus, the value of the MUT for any one year was evaluated as:

$$\text{MUT} = \frac{1}{12} \times (\text{patient-years at risk} \times \text{age-specific incidence rate}).$$

For MUT diagnosed coincidentally with the first primaries:

$$\text{MUT} = \frac{1}{12} \times (\text{number of patients} \times \text{age-specific incidence rate}).$$

For a preclinical period >12 months, such as was found for breast, an additional value must be computed substituting (age + 1) in the above equation.

### RESULTS

### *Age at first primary diagnosis*

The result of the analysis in terms of age at first primary is given in Table I, and it can be compared with those for Method 1 (Table I) and Method 2 (Table III) in the previous report (Prior & Waterhouse, 1978).

Analysis in terms of the model showed that there was a highly significant ($P <$ 0·001) excess of second primary tumours for all patients diagnosed with a first primary before the age of 60. Between the ages of 60 and 74 the level of significance was more variable. After the age of 75 the observed number (22) was not significantly different from the expected number (21·3).

Except for age groups 20–24 and 25–29, the relative risk is lower at each age than that obtained with Method 1, although the

TABLE I.—*Relative risk of a second primary breast tumour in relation to age at first primary* (*Method* 3)

| Age Group | E | O | O/E | *P* |
|---|---|---|---|---|
| 15–19 | 0·00† | 0 | — | — |
| 20–24 | 0·01 | 3 | 300·0 | *** |
| 25–29 | 0·15 | 6 | 40·0 | *** |
| 30–34 | 0·91 | 9 | 9·9 | *** |
| 35–39 | 4·25 | 28 | 6·6 | *** |
| 40–44 | 12·51 | 49 | 3·9 | *** |
| Premenopausal | 17·83 | 95 | 5·3 | *** |
| 45–49 | 19·97 | 79 | 4·0 | *** |
| 50–54 | 18·32 | 59 | 3·2 | *** |
| 55–59 | 18·83 | 52 | 2·8 | *** |
| Perimenopausal | 57·12 | 190 | 3·3 | *** |
| 60–64 | 20·17 | 33 | 1·6 | ** |
| 65–69 | 19·47 | 35 | 1·8 | *** |
| 70–74 | 15·56 | 24 | 1·5 | * |
| 75–79 | 11·40 | 10 | 0·9 | — |
| 80–84 | 6·48 | 10 | 1·5 | — |
| 85–89 | 2·58 | 2 | 0·8 | — |
| 90–94 | 0·58 | 0 | — | — |
| 95+ | 0·23 | 0 | — | — |
| Postmenopausal | 76·47 | 114 | 1·5 | *** |
| Total | 151·42 | 399 | 2·6 | |

† 1 patient "at risk".
E = Expected number of second primary tumours.
O = Observed number of second primary tumours.
* $P < 0{\cdot}05$; ** $P < 0{\cdot}01$; *** $P < 0{\cdot}001$.

level of significance has not altered except for age group 85–89, which had previously shown an excess of borderline significance ($P < 0{\cdot}05$).

In terms of the 3 age ranges, relative risk was shown, again, to decrease with increasing age at first primary, and although the values were marginally lower than those obtained by Method 1, all 3 remained highly significant ($P < 0{\cdot}001$).

*Interval between diagnoses*

Observed numbers of tumours are shown in Fig. 4, in comparison with expected numbers predicted from the model. For coincidental tumours, the expectation was 43·82, where 93 were observed, and for the whole of the first year the corresponding values were 63·27 and 146.

Over the first 5 years the pattern of expectation diverges from the linear effect shown in Fig. 2 of the previous report, and

FIG. 4.—Observed (—) and expected (- - -) numbers of second primary breast tumours and the corresponding relative risk for the first 9 years of observation (Method 3).

although there is an abrupt rise at Year 6 this is, in fact, matched by a corresponding rise in the observed number.

In Fig. 4 the relative risk over the same period is seen to rise to a peak at 3 years after the diagnosis of the first primary. After 5 years the pattern of observed and expected numbers was the same as that given in Method 1.

*Comparison of the three methods*

Table II compares the results for the first 5 years of observation; subsequent years will, of course, remain the same for each method of analysis. Taking all ages together, the relative risk computed by Method 3 lies between the values for Methods 1 and 2, and is marginally closer

TABLE II.—*Comparison of results by analytical method for the first 5 years of observation, according to age at first primary*

| Method | | All ages | Age at 1st primary 15–44 | 45–59 | 60+ |
|---|---|---|---|---|---|
| 1 | O | 301 | 71 | 142 | 88 |
| | E | 85·26 | 8·30 | 30·87 | 46·09 |
| | O/E | 3·5 | 8·6 | 4·6 | 1·9 |
| 2 | O | 212 | 65 | 107 | 33 |
| | E | 85·26 | 8·30 | 30·87 | 46·09 |
| | O/E | 2·5 | 7·8 | 3·5 | 0·7 |
| 3 | O | 301 | 71 | 142 | 88 |
| | E | 104·58 | 9·30 | 36·73 | 58·55 |
| | O/E | 2·9 | 7·6 | 3·9 | 1·5 |

to 2. A similar effect was also observed in the peri- and post-menopausal groups, but in the premenopausal group Method 2 gave a higher relative risk of 7·83 in comparison with that of 7·63 for Method 3.

A comparison of relative risk over the whole of the survey period is given in Table III. Again, Method 3 gives a value intermediate between Methods 1 and 2, both overall and for the 3 age ranges. The final values of 5·3, 3·3 and 1·5 for the pre-, peri- and post-menopausal groups, respectively, represent a relative risk of 2·6 for the whole series.

TABLE III.—*Comparison of results by analytical method for all years of observation, according to age at first primary*

| Method | | All ages | 15–44 years | 45–59 years | 60+ years |
|---|---|---|---|---|---|
| 1 | O | 399 | 95 | 190 | 114 |
| | E | 131·76 | 16·82 | 51·25 | 63·69 |
| | O/E | 3·0 | 5·6 | 3·7 | 1·8 |
| 2 | O | 310 | 89 | 155 | 66 |
| | E | 131·76 | 16·82 | 51·25 | 63·69 |
| | O/E | 2·4 | 5·29 | 3·0 | 1·0 |
| 3 | O | 399 | 95 | 190 | 114 |
| | E | 151·42 | 17·83 | 57·11 | 76·48 |
| | O/E | 2·6 | 5·33 | 3·3 | 1·5 |

### DISCUSSION

Although the concept of anticipative diagnosis has been generally recognized in the literature no numerical allowance has been made for its effect. On the basis of the method of analysis presented here, an increase in the expected number of 14·9% was demonstrated. Anticipative diagnosis has a maximal effect at first primary diagnosis, when every patient is assumed to undergo detailed examination, but with regular surveillance, a residual effect operates for several years. If every patient were to be examined at, say, yearly intervals—that is, an interval less than the duration of the preclinical period—all second primaries, would, theoretically, be discovered in the preclinical phase. In practice this does not happen, because either (i) patients are discharged from hospital attendance for follow-up because they are considered to be well enough or perhaps because they may be too old or infirm to attend, or (ii) the interval between examinations increases because of missed or delayed appointments.

We consider that the concept of the monthly unit tumour (MUT) is apt when working in the context of numbers of tumours, and it is useful both in visualizing the clinical situation and in facilitating computation by allowing manipulation within the framework of an existing method of analysis.

So many factors affect the presentation of tumours after the first diagnosis that it has not been possible to substantiate quantitatively the pattern of decline in the preclinical period. However, after many simulations with the model, it has been concluded that the peak in risk at 3 years is not artefactual (*i.e.* is not a consequence of the model) and that the linear approach gives a more acceptable pattern of risk than, say, a decline of logarithmic form.

Taking the cut-off point for hospital attendance at 5 years was a compromise, because clinical practice and patient reaction varied widely for follow-up procedure. Breast, too, poses a special problem in that the contralateral breast is easily examined without causing undue anxiety in the patient, with the result that surveillance for this site is greater than that for other possible second primary sites.

The preclinical period, in this context,

TABLE IV.—*Relative risk in relation to the length of the preclinical period.* (*Unsmoothed data for* 6 *years of observation.*)

| | Age at 1st primary (years) | | | | | |
|---|---|---|---|---|---|---|
| | 15–44 | | 45–59 | | 60+ | |
| | Preclinical period (months) | | Preclinical period (months) | | Preclinical period (months) | |
| Year from 1st primary | 16·0 | 3·4* | 16·0 | 16·7* | 16·0 | 25·1* |
| Coincidental | 2·1 | 8·3 | 2·6 | 2·5 | 1·9 | 1·2 |
| 1 | 8·4 | 7·1 | 4·5 | 4·5 | 0·9 | 1·3 |
| 2 | 12·6 | 9·8 | 5·7 | 5·8 | 0·9 | 1·1 |
| 3 | 16·7 | 12·8 | 5·8 | 5·9 | 2·2 | 2·8 |
| 4 | 5·9 | 4·6 | 3·3 | 3·4 | 2·2 | 2·8 |
| 5 | 10·0 | 7·8 | 3·3 | 3·3 | — | — |
| 6 | 7·3 | 7·3 | 2·5 | 2·5 | 1·3 | 1·3 |

* Value obtained from intercepts in the regression analysis (see Part I).

encompassed all detectable but not yet diagnosed tumours, and the value of 16 months for the duration of the preclinical phase again represents a typical figure with a wide variation. This value falls, however, between two published estimates of 20 months (Hutchison & Shapiro, 1968) based on mammographic examination and 10 months (Bross *et al.*, 1968) which was a hypothetical value based on the assumption of logarithmic growth of tumours.

Several hypotheses concerning the level of risk at first examination were investigated, and an extreme evaluation, assuming an age-differential pattern, is given in Table IV for comparison with that for a constant preclinical period. We found, nevertheless, that for a wide range of values the apparent peak in risk in the 3rd year remained. A 5-fold risk at this point was obtained using a constant value for the preclinical period.

An aetiological explanation of the peak is not immediately obvious. If multiple tumours are the result of the same carcinogenic challenge, a lag of 3 years between their maturation is difficult to explain. It may be, however, that the peak is related to an event other than the induction of the first primary. There is considerable evidence for multiple transformation of the cells throughout the breast tissue: in one study (Qualheim, 1957) 54% of breasts removed were found to have multiple foci of malignant transformation, and in another report (Bloom, 1968) the change was considered to be almost certainly bilateral. This would suggest that not all malignant foci progress to clinical cancer, either because they are being held in check by the immune system or because of slow growth, or, even, because they may spontaneously regress, but once one focus has progressed to a critical size in relation to the state of immune surveillance, it may be that a second focus can break away from homoeostatic control and come to diagnosis at an interval from the first primary.

However, another common point in time could be diagnosis of (and possibly treatment to) the first primary, a time which might again represent a period of immune stress. A short period of immune depression has been shown to occur after major surgery (Cochran *et al.*, 1972) and radiotherapy also has come under close scrutiny for the same reason (Bond, 1967). Although the surgical effect was transitory (6–22 days) and the effect of radiotherapy localized, they may be sufficient to allow already transformed cells to escape from control and proceed to express their malignant potential. The cause of the peak is not clear, but it would appear from our results that its effect on risk is more pronounced in younger women.

Although the data from the bilateral breast survey have been used here to present the model, the effect of anticipatory diagnosis is a common problem of many epidemiological investigations in which clinical intervention for one condition influences the presentation of a second. Evaluation of associations between cancer and other diseases or between non-malignant conditions, for instance, would require similar adjustments.

The model is, perhaps, only a simple representation of a complex situation, but it could be refined if more detailed information were available. Nevertheless with such large numbers some generalization is acceptable. Use of the model for analyses of other site pairs will, it is hoped, provide further justification for an approach which allows the use of all available information, thus avoiding the need to make arbitrary exclusions.

The survey of multiple primary malignant tumours is supported by the Cancer Research Campaign.

### REFERENCES

BERG, J. W. (1967) The incidence of multiple primary cancers. I. Development of further cancers in patients with lymphomas, leukaemia and myeloma. *J. Natl Cancer Inst.*, **38**, 741.

BLOOM, H. J. G. (1968) Survival of women with untreated breast cancer: Past and present. In *Prognostic Factors in Breast Cancer*. Eds Forrest & Kunkler. Edinburgh: Livingstone. p. 23.

BOND, W. H. (1967) The influence of various treatments on survival rates in cancer of the breast. In *The Treatment of Carcinoma of the Breast*. Ed. Jarrett. Amsterdam: Excerpta Medica Foundation. p. 24.

BROSS, I. D. J., BLUMENSON, E., SLACK, N. H. & PRIORE, R. L. (1968) A two-disease model for breast cancer. In *Prognostic Factors in Breast Cancer*. Eds Forrest & Kunkler. Edinburgh: Livingstone. p. 288.

COCHRAN, A. J., SPILG, W. G. S., MACKIE, M. & THOMAS, C. E. (1972) Postoperative depression of tumour-directed cell-mediated immunity in patients with malignant disease. *Br. Med. J.*, iv, 67.

GREENBERG, R. A. (1963) The occurrence of multiple primary cancers in Connecticut 1935–1954. *Conn. Health Bull.*, **77**, 257.

HUTCHISON, G. B. & SHAPIRO, S. (1968) Lead time gained by diagnostic screening. *J. Natl Cancer Inst.*, **41**, 665.

PRIOR, P. (1974) The statistical status of coincidental tumours in surveys of multiple primary cancers. In *Multiple Primary Malignant Tumours*. Ed. Severi. Perugia: Division of Cancer Research. p. 201.

PRIOR, P. & WATERHOUSE, J. A. H. (1978) Incidence of bilateral tumours in a population-based series of breast-cancer patients. I. *Br. J. Cancer*, **37**, 620.

QUALHEIM, R. E. & GALL, E. A. (1957) Breast cancer of multicentric origin. *Cancer*, **10**, 460.

ROBBINS, G. F. & BERG, J. W. (1964) Bilateral primary breast cancers. *Cancer*, **17**, 1501.

SCHOENBERG, B. S., GREENBERG, R. A. & EISENBERG, H. (1969) Occurrence of certain multiple primary cancers in females. *J. Natl Cancer Inst.*, **43**, 15.

SCHOENBERG, B. S. & CHRISTINE, B. W. (1974) The Association of neoplasms of the colon and rectum with primary malignancies of other sites. *Am. J. Proctol.*, **25**, 41.

SCHOTTENFELD, D. & BERG, J. (1971) Incidence of multiple primary cancers, IV. Cancers of the female breast and genital organs. *J. Natl Cancer Inst.*, **46**, 161.

VERONESI, U., RILKE, F., SALVADORI, B., DEL VECCHIO, M. & ZANOLLA, R. (1974) Bilateral cancer of the breast. In *Multiple Primary Malignant Tumours*. Ed. Severi. Perugia: Division of Cancer Research. p. 337.